# A Comprehensive AI Framework for Superior Diagnosis, Cranial Reconstruction, and Implant Generation for Diverse Cranial Defects

**DOI:** 10.3390/bioengineering12020188

**Published:** 2025-02-16

**Authors:** Mamta Juneja, Ishaan Singla, Aditya Poddar, Nitin Pandey, Aparna Goel, Agrima Sudhir, Pankhuri Bhatia, Gurzafar Singh, Maanya Kharbanda, Amanpreet Kaur, Ira Bhatia, Vipin Gupta, Sukhdeep Singh Dhami, Yvonne Reinwald, Prashant Jindal, Philip Breedon

**Affiliations:** 1 University Institute of Engineering and Technology, Panjab University, Chandigarh 160014, India; mamtajuneja@pu.ac.in (M.J.); sishaan380@gmail.com (I.S.); adityapoddar885@gmail.com (A.P.); nitincomp07@gmail.com (N.P.); aparnagoel444@gmail.com (A.G.); agrimasian10@gmail.com (A.S.); pankhuri0307@gmail.com (P.B.); gurzafarsingh@gmail.com (G.S.); kharbandamaanya@gmail.com (M.K.); amanpreet0303kaur@gmail.com (A.K.); irabhatia2004@gmail.com (I.B.); 2 Department of Neurosurgery, Government Medical College and Hospital, Sector 32, Chandigarh 160032, India; vipingupta07@gmail.com; 3 Department of Mechanical Engineering, National Institute of Technical Teachers Training and Research, Chandigarh 160019, India; ssdhami@nitttrchd.ac.in; 4 School of Science and Technology, Department of Engineering, Nottingham Trent University, Nottingham NG1 4FQ, UK; yvonne.reinwald@ntu.ac.uk; 5 Medical Technologies Innovation Facility, School of Science and Technology Nottingham Trent University, Nottingham NG1 4FQ, UK

**Keywords:** artificial intelligence, cranial reconstruction, computer-aided diagnosis, deep learning, CT imaging, preprocessing, augmentation

## Abstract

Cranioplasty enables the restoration of cranial defects caused by traumatic injuries, brain tumour excisions, or decompressive craniectomies. Conventional methods rely on Computer-Aided Design (CAD) for implant design, which requires significant resources and expertise. Recent advancements in Artificial Intelligence (AI) have improved Computer-Aided Diagnostic systems for accurate and faster cranial reconstruction and implant generation procedures. However, these face inherent limitations, including the limited availability of diverse datasets covering different defect shapes spanning various locations, absence of a comprehensive pipeline integrating the preprocessing of medical images, cranial reconstruction, and implant generation, along with mechanical testing and validation. The proposed framework incorporates a robust preprocessing pipeline for easier processing of Computed Tomography (CT) images through data conversion, denoising, Connected Component Analysis (CCA), and image alignment. At its core is CRIGNet (Cranial Reconstruction and Implant Generation Network), a novel deep learning model rigorously trained on a diverse dataset of 2160 images, which was prepared by simulating cylindrical, cubical, spherical, and triangular prism-shaped defects across five skull regions, ensuring robustness in diagnosing a wide variety of defect patterns. CRIGNet achieved an exceptional reconstruction accuracy with a Dice Similarity Coefficient (DSC) of 0.99, Jaccard Similarity Coefficient (JSC) of 0.98, and Hausdorff distance (HD) of 4.63 mm. The generated implants showed superior geometric accuracy, load-bearing capacity, and gap-free fitment in the defected skull compared to CAD-generated implants. Also, this framework reduced the implant generation processing time from 40–45 min (CAD) to 25–30 s, suggesting its application for a faster turnaround time, enabling decisive clinical support systems.

## 1. Introduction

Cranioplasty is a vital surgical procedure aimed at repairing skull defects caused by brain tumour removal, traumatic injury, or decompressive craniectomy. It serves dual purposes: restoring the skull’s structural integrity and improving the patient’s appearance [1]. Successful cranioplasty depends heavily on the precise design of custom implants tailored to the patient’s unique skull morphology. Traditional implant design involves high-resolution CT scans of the damaged skull, which are then used in conjunction with CAD software to manually create 3D models for implants. Although effective, this process is time-consuming, labour-intensive, and requires significant expertise.

Recent technological advancements, particularly in 3D printing, have significantly improved cranial implant design and manufacturing. Preoperative CT scans are now utilized for designing implants, which are then 3D-printed using biocompatible materials such as titanium, porous polyethylene, or polyether-ether-ketone (PEEK) [2,3]. These innovations have enhanced the flexibility and precision of implant customization, allowing for better integration with the patient’s skull. Alternatively, bone cement enhanced with antibiotics can be cast into 3D-printed moulds to produce implants [4]. However, challenges remain, particularly regarding the accuracy of tissue segmentation from CT data, which is essential for creating precise implants. Moreover, traditional methods often require significant manual input and expertise [5], leaving room for potential improvements in automation and precision.

Advancements in Artificial Intelligence (AI) and deep learning (DL) have the potential to revolutionize cranial defect reconstruction and implant design. AI algorithms, particularly those based on deep learning, offer robust tools for automating and improving multiple stages of the cranioplasty process, including image segmentation, defect identification, and 3D reconstruction. These AI techniques significantly reduce manual labour, increase reproducibility, and improve the customization of implants. By handling large and complex datasets, AI models can achieve high accuracy and speed, thus enhancing their adaptability to a wide range of patient-specific conditions, including asymmetry and rare defect types.

### 1.1. AI Framework for Superior Diagnosis

One of the most significant applications of AI in cranial defect reconstruction is in image processing and diagnosis. Conventional methods rely heavily on manual segmentation and defect identification from CT scans, which are prone to human error and variability. AI-powered algorithms, particularly Convolutional Neural Networks (CNNs), offer the ability to handle high-resolution 3D medical data more efficiently and accurately. For instance, Li et al. [6] applied CNNs to address the high-resolution nature of 3D medical scans, framing cranial defect reconstruction as a 3D volumetric shape completion task. By training the model on synthetically generated skull defects, they achieved superior performance compared to traditional CAD-based methods. The use of an encoder–decoder network with skip connections allowed for the preservation of vital spatial information, ensuring more accurate defect restoration.

Building on this foundation, Wu et al. [7] introduced a sophisticated 3D deep learning framework aimed at automating the generation of cranial implant geometries directly from defective skull models. This approach overcame the limitations of symmetry-based methods traditionally used in cranial reconstruction. By employing 3D autoencoder architecture, their model was able to predict complete implant geometries from incomplete skull data, improving both accuracy and computational efficiency. This development represented a significant progression in automating cranial reconstruction and implant design. Further advancing this technology, Li et al. [8] integrated a deep learning model into a cloud-based platform known as Studierfenster, streamlining two crucial tasks in cranioplasty: defect segmentation in post-operative CT scans and implant generation.

### 1.2. Cranial Reconstruction

The next critical stage in cranioplasty is cranial reconstruction, which involves accurately restoring the skull to its original shape. Deep learning methods, particularly those that leverage autoencoders and other neural network architectures, have shown great promise in automating this step. Kodym et al. [9] explored the use of Generative Adversarial Networks (GANs) integrated with CNNs to improve cranial defect reconstruction. By combining adversarial loss functions and latent vector injections, their model was able to handle more complex defects, thus increasing its flexibility and generalization capabilities. Wodzinski et al. [10] employed modified U-Net architectures to enhance the accuracy of defect restoration and implant modelling. This approach demonstrated how neural networks could be tailored to handle the specific challenges posed by cranial defects. Li et al. [11] further extended his work by implementing an autoencoder within the Medical Open Network for Artificial Intelligence (MONAI) framework, broadening the scope of AI models to handle both cranial and facial defects with increased robustness.

Another significant advancement was made by Wodzinski et al. [12], who developed a self-supervised deep deformable masked autoencoder to address cranial defect reconstruction. This novel method used masked autoencoders to randomly remove portions of input skull images, reconstructing the missing parts. This increased the variability in the training data and improved the model’s ability to generalize across diverse defect types. Their architecture, based on a Residual 3D UNet backbone, achieved superior performance, particularly in handling cranial defects with high accuracy and generalizability.

### 1.3. Implant Generation for Diverse Cranial Defects

Implant generation is another crucial aspect of cranioplasty that has been transformed by AI. The process of designing custom implants that precisely fit the reconstructed skull remains a challenging task. Advances in deep learning, particularly through generative models, have opened new avenues for the generation of custom implants. Matzkin et al. [13] introduced the DE-UNet and DE-Shape-UNet models, which incorporated shape priors to improve robustness in reconstructing cranial implants. These models are particularly effective in handling rare or complex defects that are not commonly seen in the training data. Kwarciak and Wodziński et al. [14] discussed three volumetric variations of deep generative models: a Wasserstein Generative Adversarial Network with Gradient Penalty (WGAN-GP), a hybrid WGAN-GP with Variational Autoencoder Pretraining (VAE/WGAN-GP), and an Introspective Variational Autoencoder (IntroVAE), which generated synthetic skulls with multiple defects. This approach enhanced the segmentation process by providing a more diverse and realistic training dataset, thereby improving the performance of cranial defect reconstruction. These studies highlighted the importance of data augmentation in enhancing the accuracy and robustness of cranial implant designs for more effective solutions.

Another significant innovation in this area was the use of ensemble learning by Yang et al. [15], which tackled the challenge of overfitting in 3D deep learning models for skull reconstruction by slicing 3D skull volumes into 2D planes and employing both CNN and Recurrent Neural Network (RNN) models to predict implants. The ensemble learning method significantly improved implant prediction accuracy, highlighting the benefits of multi-axial slicing and ensemble strategies in cranial implant design. Furthermore, Resmi et al. [16] demonstrated the integration of 3D U-Net with transformers for automatic skull shape completion, offering improvements in both accuracy and processing speed. However, this approach used lower-resolution images (64 × 64 × 64), which may limit its applicability in clinical settings that typically require higher-resolution data. Li et al. [17] employed a Statistical Shape Model (SSM) combined with Principal Component Analysis (PCA) for reconstructing large and complex cranial defects, achieving robust performance, particularly in clinical cases. The approach demonstrated strengths in mathematical shape representation, though it exhibited suboptimal performance on synthetic defects compared to CNN-based methods, highlighting the need for advancements in deep learning network quality and registration accuracy for broader clinical applications. Table 1 provides a comparative analysis of existing AI-based cranial reconstruction techniques, highlighting their methodologies, merits, and demerits.

It is evident from Table 1 that advancements in AI and deep learning technologies have significantly transformed cranial defect reconstruction, offering faster, more accurate, and more efficient solutions compared to traditional methods. However, despite these improvements, several challenges remain, including improving the generalizability of AI models to handle complex defects, the need for manual post-processing, the variability in outcomes based on defect size and location, and production of high-resolution cranial implants. These challenges highlight the ongoing need for research aimed at addressing these gaps and further enhancing the accuracy and efficiency of AI-driven cranial reconstruction methods.

### 1.4. Materials for Cranial Implants

Equally important in cranioplasty is the selection of an appropriate biomaterial for the custom implants. The choice of biomaterial influences the implant’s integration with surrounding tissue, its mechanical strength, and the patient’s recovery. Titanium and PEEK are two widely used materials for cranial implants, due to their favourable biocompatibility and mechanical properties [18]. Titanium provides excellent strength but can cause stress shielding, hindering bone integration. PEEK, on the other hand, is bioinert and lightweight, with mechanical properties similar to human bone, making it an ideal choice for cranial implants [19]. PEEK also demonstrates compatibility with various imaging modalities, including X-ray, CT, ultrasound, and Magnetic Resonance Imaging (MRI). PEEK’s superior mechanical properties, including its elasticity, thermal stability, and rigidity, contribute to better bone integration and faster recovery [20,21,22]. Compared to titanium, PEEK improves brain function, cosmetic outcomes, and reduces deformation risks under pressure, enhancing safety in replacement surgeries. Its superior mechanical properties, thermal stability, rigidity, and low moisture absorption make it a more effective option for cranioplasty [23,24]. With a lower failure rate and minimal stress shielding, PEEK ensures better implant fit, stability, and strength while minimising aesthetic variations. A 3D reconstruction approach using PEEK further streamlines implant adjustments and enhances procedural efficiency [25,26,27].

### 1.5. Overview of the CRIGNet Framework

This paper introduces CRIGNet, a novel AI-driven framework for cranial reconstruction and implant generation. CRIGNet leverages advanced deep learning algorithms and PEEK as the implant material to optimize cranial reconstruction workflows and produce more accurate, personalized implants for patients. The framework integrates real-time CT data acquisition, advanced preprocessing, and automated reconstruction and implant generation to improve precision and processing time. By incorporating diverse defect generation techniques and evaluating model performance through comprehensive validation, CRIGNet aims to set a new standard in personalized cranial implants and improve surgical outcomes with the following key innovations:

1. Versatile AI Framework for Cranial Reconstruction: CRIGNet establishes an end-to-end framework integrating real-time CT data acquisition, advanced preprocessing, and automated reconstruction and implant generation, addressing challenges in manual and time-intensive CAD-based workflows [6,7,8,9,10].
2. Robust Preprocessing Pipeline: The pipeline incorporates thresholding, denoising, Connected Component Analysis (CCA), and image alignment to ensure high-quality input data, mitigating inaccuracies in defect segmentation [6,8].
3. Advanced Defect Generation Methods: CRIGNet introduces diverse defect generation across five skull regions, enhancing training with varied shapes of defect cases, a significant improvement over existing models [7,10,13].
4. Innovative AI Architecture: CRIGNet employs a novel deep learning architecture tailored for accurate cranial defect reconstruction and implant generation. This approach handles a wide range of defect geometries effectively, outperforming traditional and existing AI methods [9,10,11,12,13].
5. Comprehensive Evaluation of Model Performance: CRIGNet is rigorously compared against state-of-the-art AI and conventional models, demonstrating superior performance across accuracy, processing efficiency, and defect generalizability [6,11,28,29,30].
6. Mechanical Validation of Implants: The framework includes finite element analysis (FEA) to validate implant accuracy, strength, and load-bearing capacity, addressing gaps in biomechanical assessments from prior studies.
7. Augmented Dataset Creation: The study augments the MUG500+ dataset, consisting of 500 skulls. A total of 360 healthy skulls were extracted for further processing and injected with defects to form 2160 data pairs with diverse geometries. This dataset enables robust training and testing, overcoming limitations in dataset variability observed in prior works [11].
8. Material Integration for Enhanced Clinical Outcomes: Leveraging PEEK material for implant generation, CRIGNet combines AI-driven precision with biocompatibility and strength, providing better clinical outcomes compared to titanium-based implants [18,19,20].

## 2. Materials and Methods

**Figure 1:**
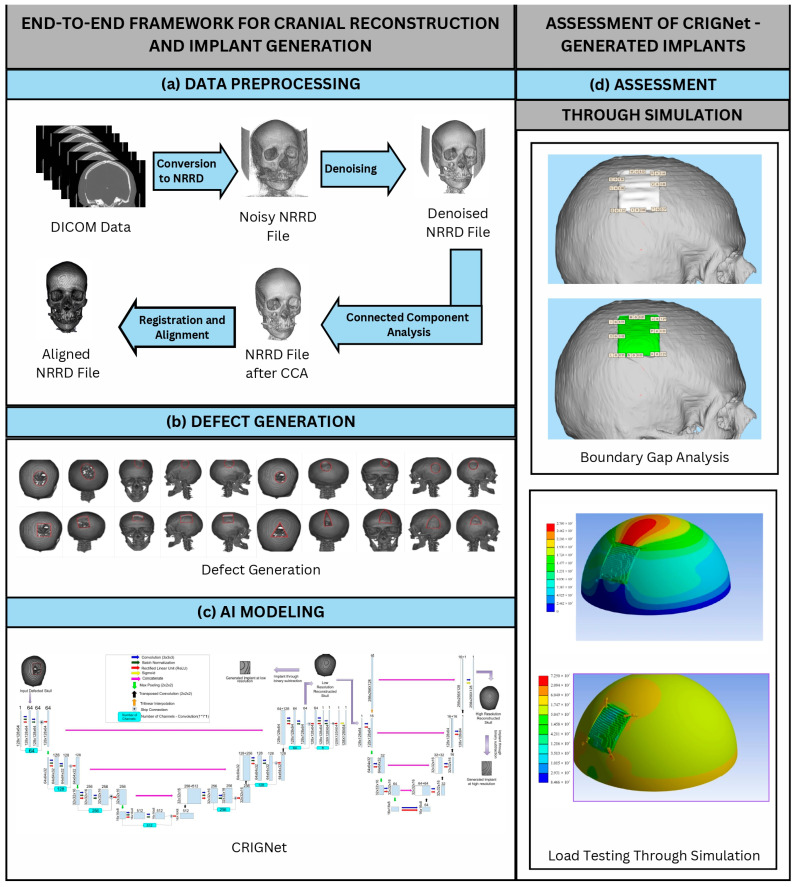
Proposed end-to-end framework for cranial reconstruction and implant generation and its assessment: (**a**) data preprocessing, (**b**) defect generation, (**c**) AI modelling, and (**d**) assessment of CRIGNet generated implants.

This section of the paper begins with a description of the healthy skull MUG500+ [31] dataset acquired for the research. The sections following it provide an overview of the proposed end-to-end framework for cranial reconstruction and implant generation, graphically summarised in Figure 1. Figure 1a shows the successively applied preprocessing steps, followed by the augmentation of the dataset, performed as shown in Figure 1b through defect generation. Next, Figure 1c shows an outline of the architecture of the proposed model CRIGNet and Figure 1d summarises the assessment of CRIGNet-generated implants, which is implemented through the simulation of the boundary gap measurement and load testing.

### 2.1. Dataset Description

A skull database called MUG500+ [31] was used in this study, derived from high-resolution head CT scans collected and curated by the Medical University of Graz, Austria. This dataset had 500 complete skulls, encompassing cranial, mandibular, and maxillofacial bones along with associated structures. The origin of this dataset is the clinical imaging repository of the Medical University of Graz. Out of the 500 available skulls, 396 were deemed suitable for further processing for our task. The remaining 104 skulls were excluded due to issues such as incomplete imaging data (e.g., single slices), the presence of artefacts like oxygen masks, or excessive noise that compromised their structural integrity and usability for model training and evaluation. The final usable dataset includes 396 high-resolution skulls stored in NRRD format, with dimensions of 512 × 512 × Z, where Z represents the number of axial slices.

### 2.2. Proposed End-to-End Framework for Cranial Reconstruction and Implant Generation

#### 2.2.1. Preprocessing Pipeline

Preprocessing steps were applied to the raw dataset to obtain a consistent and standardized dataset. The preprocessing pipeline began with converting Digital Imaging and Communications in Medicine (DICOM) images to Nearly Raw Raster Data (NRRD) format to enable flexibility in processing and integration with various software packages. This step is part of a generalized pipeline that ensures applicability to real-time input images in DICOM format, though the acquired MUG500+ dataset was already in NRRD format and thresholded. The next step involved denoising, which was performed using the Median filter to remove Gaussian, salt-and-pepper, speckle, and visual noise commonly found in CT scan images [32,33]. Following this, CCA was applied to eliminate CT artefacts such as supporting plates and positioning aids, ensuring that only the skull morphology remained for further processing [34].

Registration and alignment techniques were then applied to correct the orientation of 36 misoriented skull files, allowing accurate defect generation in the desired regions. Finally, the images were down sampled to 256 × 256 × 128 before defect generation, and subsequently rescaled to 128 × 128 × 64 to optimize computational efficiency during model training for reconstruction.

#### 2.2.2. Defect Generation

Since the original acquired dataset consisted of complete healthy skulls, it was necessary to simulate a variety of cranial defects on it. By introducing artificial defects of different shapes and sizes in key regions, this process not only increased the size and diversity of the dataset but also ensured that the AI model is trained to diagnose patterns and reconstruct a wide range of potential defects. This step was critical for creating accurate, patient-specific cranial implants in the subsequent stages of the framework, enabling the AI model to perform precise reconstructions based on diverse defect patterns.

**Figure 2:**
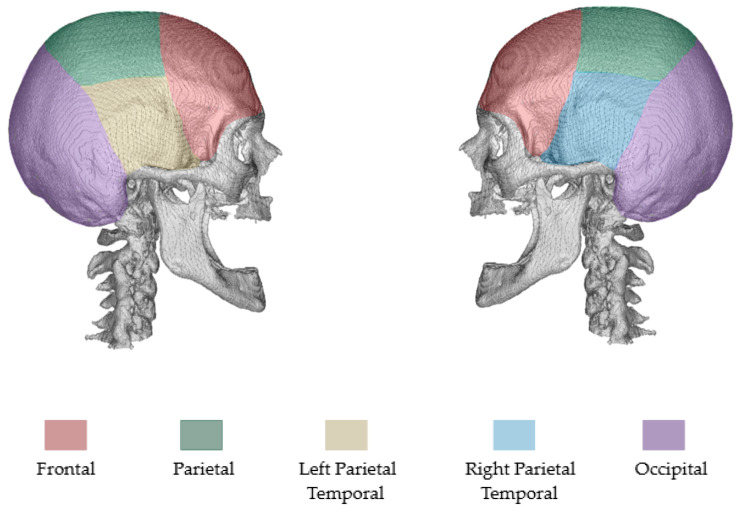
Regions for defect generation.

The process included identifying key regions to introduce defects: parietal [35], left parietal temporal [35], right parietal temporal [35], frontal [35], and occipital (in this paper, the regions of defects, including parietal, left parietal temporal, right parietal temporal, frontal, and occipital, will be referred to as follows for easier understanding: top, left, right, front, and back, respectively). Figure 2 illustrates the regions mentioned above, using coloured representations on an NRRD file from the MUG500+ dataset.

The raw skull dataset was augmented by dividing it into five primary regions: top, left, right, front, and back. Various defect types—spherical [36], cubical [36], cylindrical, and triangular prism—were then introduced into each of these regions. By systematically adding these defects to each region, a total of 20 types of defects were created, i.e., in each of the 5 regions, there are defects of 4 shapes: spherical, cubical, cylindrical, and triangular prisms (used interchangeably as triangular); this results in 5 × 4 = 20 cases.

A general method for creating defects in 3D models involved specifying the geometric parameters for the desired shape, including sphere (radius and position), cube (dimensions and position), triangular prism (base, height, and centre), and cylinder (radius, height, and centre). A 3D grid representing the model’s coordinates was generated, and points within the boundaries of the defined shape were identified to create a corresponding mask. These masks were applied to the original 3D model, setting the defect points to zero to simulate the defect. Separate implant masks were also generated to represent the defected regions, enabling precise modifications for each shape.

#### 2.2.3. Proposed Model CRIGNet

**Figure 3:**
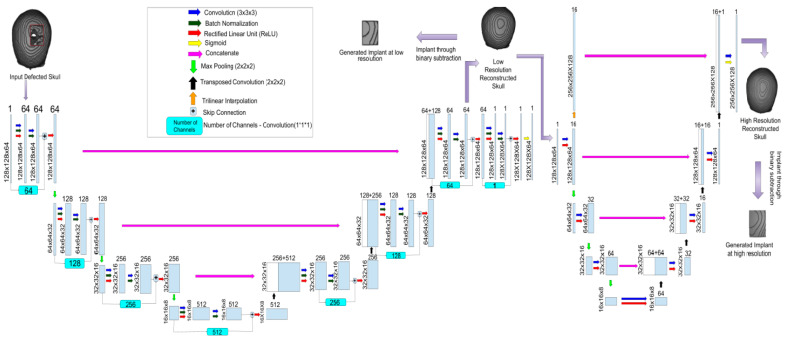
Architecture of the proposed AI model CRIGNet.

Defect generation was followed by the training of the AI model for the reconstruction of cranial regions of the skulls, enabling implants to be generated given the input of a defected skull. This section of the paper, therefore, proposes a novel AI-based model for the cranial reconstruction of defected skulls and implant generation. The architecture diagram of the proposed AI model, named Cranial Reconstruction and Implant Generation Network (CRIGNet), is presented in Figure 3. It primarily performs two tasks: cranial reconstruction to produce a lower-resolution output, followed by resolution enhancement to produce a final reconstructed skull of higher resolution. A low-resolution implant can be generated after the reconstruction network through Boolean subtraction, while for a higher-resolution implant, the reconstructed skull is passed through the resolution enhancement network, after which Boolean subtraction is carried out.

The CRIGNet architecture leverages a ResUNet-based [37] design for cranial reconstruction and implant generation. The reconstruction network processes defected skull images of size (128, 128, 64) with one channel. Its encoder–decoder structure includes four encoder layers using residual blocks for feature extraction, batch normalisation [38] for stable training, ReLU activation [39], and Max-Pooling for down sampling. The decoder mirrors this structure, with three decoder layers using residual blocks and Conv3DTranspose for up sampling. The final decoder layer employs sigmoid activation [40] to normalise the output to a range of 0 to 1, producing a reconstructed image of varying sizes (128, 128, 64) with one channel. Skip connections between corresponding encoder and decoder layers preserve spatial information. Implant generation is performed by subtracting the defected skull from the reconstructed output using Boolean subtraction.

The resolution enhancement network further processes the reconstructed output to generate a higher-resolution image. This network follows an encoder–decoder structure with 3D convolutions, ReLU activation, and Max-Pooling in the encoder. The decoder uses Conv3DTranspose for up sampling and integrates interpolated pathways to produce an image of varying sizes (256, 256, 128) with 17 channels. A final 3D convolution, combined with sigmoid activation, outputs a high-resolution image with one channel. Boolean subtraction integrated into the network ensures seamless implant generation.

The reconstruction network containing 26,531,874 trainable parameters was trained for 200 epochs, while the resolution enhancement network with 465,225 trainable parameters was trained for 50 epochs. Both networks used the Adam optimizer [41], dice loss, and metrics such as the Dice Similarity Coefficient (DSC), Jaccard Similarity Coefficient (JSC), Hausdorff distance (HD), precision, recall, and specificity, with an initial learning rate of 0.001 and a batch size of 2.

CRIGNet leverages ResUNet architecture, batch normalisation, ReLU, and sigmoid activation to accurately diagnose defect patterns based on shapes and regions; this ensures precise cranial reconstruction and implant generation. The implants produced are referred to as “CRIGNet-generated implants”.

### 2.3. Assessment of CRIGNet-Generated Implants for Suitable Fitment: Geometrical Accuracy and Mechanical Strength

The fitment of these implants within the defected cranial cavity needs to be analysed and compared with the implants generated using conventional CAD-based methods, for a comprehensive validation of this novel CRIGNet framework.

The model for the implant fitment with the cranial defect was generated to evaluate the geometric accuracy. Finite element analysis (FEA), using ANSYS 15 2022 R2, was conducted to analyse the overall mechanical strength against failure subjected to external loadings, for a PEEK-based implant material.

The assessment involved geometric analysis, based upon implant edge gap measurements and overall mesh analysis, to determine the fitment and coverage of the implants. Implant designs that result in lower induced stresses within the implant material are preferred for safety against failures. For this reason, FEA has been used to further study the improved performance of the implant design for lightweight, bone-like materials such as PEEK. The complete processing time for the implant generation was obtained and compared for both AI and CAD methods.

## 3. Results

This section provides the quantitative performance details of methods, which are applied as per Section 2. It begins with details of the experimental setup used, followed by a visual evaluation of the preprocessing pipeline. Next, the defect generation techniques used to augment the dataset are analysed, emphasising how they contributed to a more diverse and robust training set. The performance of CRIGNet is then assessed across various skull regions, highlighting its ability to reconstruct defects with precision. A comparative performance analysis is provided, assessing CRIGNet’s results against those of existing models to assess its superiority in accuracy and efficiency.

The section concludes with a comprehensive validation of the implants generated by CRIGNet. Mechanical testing is performed to evaluate the accuracy, strength, and load-bearing capacity of the implants, providing insights into their practical applicability in clinical settings. Finally, an analysis of the time efficiency is presented.

### 3.1. Experimental Setup

The experiments were conducted on HP Z840 Workstation (HP Inc., Palo Alto, CA, USA) with an Intel Xeon E5-2650 V4 CPU (48 cores, 2.20 GHz), 256 GB RAM, and an Nvidia RTX3090 GPU (24 GB VRAM), with additional support from an NVIDIA DGX Station A100 (Dell Technologies, Round Rock, TX, USA) (4 × A100 GPUs, 320 GB GPU memory, 2 TB RAM). The workflow included data augmentation and preprocessing, defect generation, CRIGNet deployment, and comparative analyses focusing on geometric accuracy, FEA, and time efficiency.

### 3.2. Preprocessing Results

**Figure 4:**
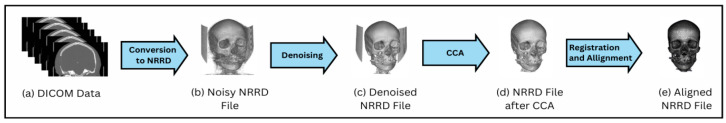
Results of various preprocessing steps: (**a**) RAW DICOM data, (**b**) noisy NRRD data found in MUG500+ dataset, (**c**) denoised NRRD file containing extraneous artefacts, (**d**) clean NRRD file with artefacts removed through CCA, and (**e**) aligned NRRD file.

The preprocessing steps enabled the refinement of the MUG500+ dataset, which was already provided in the NRRD format, and thresholded into a clean and consistent dataset for model training. Figure 4 illustrates the results of each preprocessing step applied to the raw data, as detailed in Section 2.2.1. For real-time applications involving DICOM datasets, the initial step (Figure 4a) would involve converting raw DICOM data into the NRRD format. However, for this study, the pipeline began with the MUG500+ dataset, already in NRRD format and thresholded, as shown in Figure 4b. Figure 4c shows the denoised NRRD file after applying the Median filter, characterized by the removal of salt-and-pepper noise. Following this, Figure 4d displays the NRRD file after the application of Connected Component Analysis (CCA), which successfully eliminated extraneous artefacts, retaining only the cranial region of interest. To ensure consistent defect generation, the alignment step was applied to correct image orientations, resulting in the orientation shown in Figure 4e. Finally, the down sampling of the images optimized their resolution, providing a uniform dataset for efficient model training and reconstruction. These preprocessed files were then utilized for defect generation, ensuring a consistent and reliable input for the subsequent sections of the model.

### 3.3. Data Augmentation by Defect Generation

**Figure 5:**
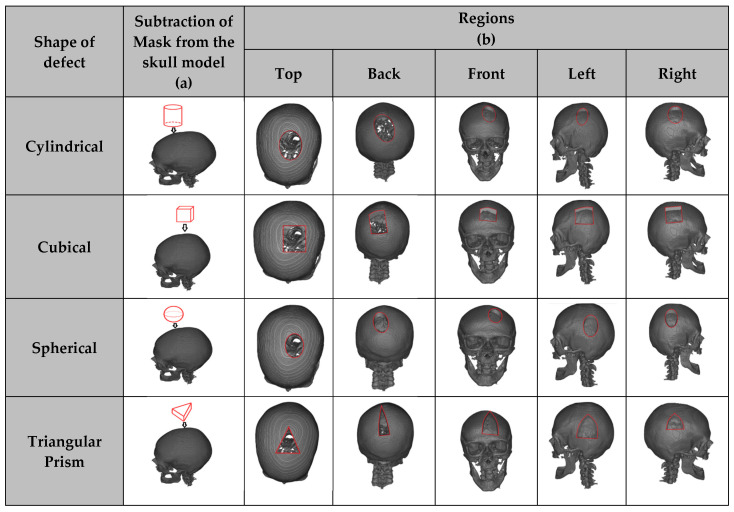
Visualisations of the various defect types created using four defect mask shapes in the five identified regions on a representative case from the MUG500+ dataset. (**a**) Masks, (**b**) Regions.

Exhaustive data augmentation through defect generation was performed to ensure that the dataset captures a wide variety of defect shapes and locations, thereby increasing the model’s generalizability. This process expanded the dataset size from 396 NRRD files to a total of 2160 skull samples. Defects were injected into five identified anatomical regions: left parietal, right parietal, occipital, parietal temporal, and frontal. Healthy skull images from the MUG500+ dataset were evenly divided into five subsets, each corresponding to one of the defect regions, with 72 skull images per subset. For each region, four defect types (cylindrical, spherical, cubic, and triangular prism) were generated, resulting in 1440 unique training samples (seventy-two skulls × four defect types × five regions). For the testing dataset, defects were created on the remaining thirty-six skulls across all five regions and four defect types, generating 720 unique testing samples (thirty-six skulls × four defect types × five regions). Figure 5 provides a detailed visualisation of the defect generation process, showcasing the different defect shapes and regions as explained in Section 2.2.2. Figure 5a demonstrates the injection of the four different defect shapes (cylindrical, spherical, cubic, and triangular prism) into the healthy skull dataset, with the red wireframe representing the shape of each defect. Figure 5b displays the resulting twenty defected skulls, covering regions such as the top, back, front, left, and right.

### 3.4. CRIGNet-Based Reconstruction and Implant Generation

**Figure 6:**
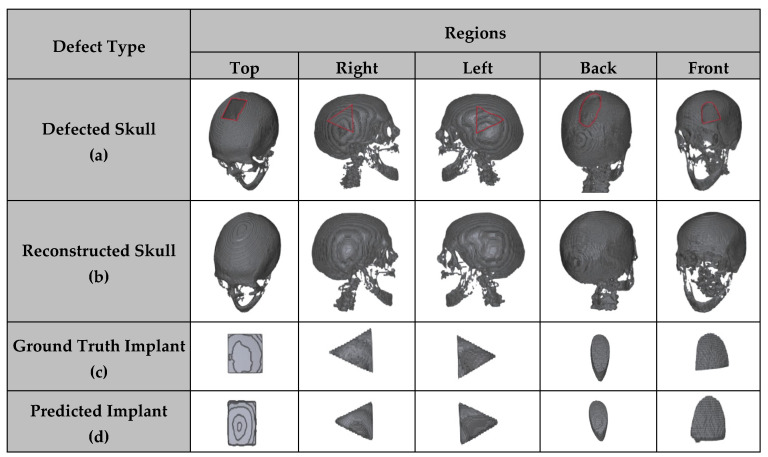
Visual results of the CRIGNet-based reconstruction and implant generation across different regions on five arbitrarily chosen test cases: (**a**) defected skull (**b**), reconstructed skull, (**c**) ground truth implant, and (**d**) predicted implant.

The proposed model, CRIGNet, successfully reconstructed the cranial region from defected skulls and generated the corresponding implants, yielding satisfactory results. Figure 6 provides a visual assessment of CRIGNet’s performance by presenting visualisations of five arbitrarily selected defected skull cases. In Figure 6a, the defected skulls are shown, followed by the corresponding reconstructions performed by CRIGNet in Figure 6b. Figure 6c presents the ground truth implants, while Figure 6d shows the implants generated by CRIGNet. A close inspection of both the reconstructions and the generated implants reveals their close resemblance to the ground truth geometry, further supporting the quantitative results presented in the subsequent sections.

#### Performance Analysis of CRIGNet-Based Cranial Reconstruction

A diverse set of performance metrics was utilized to comprehensively evaluate the model’s accuracy and robustness in cranial reconstruction tasks. The metrics used were DSC [6], JSC [6], HD [42], precision [6], recall [6], and specificity [43].

- DSC: Measures the overlap between predicted and ground truth regions, ranging from 0 (no overlap) to 1 (perfect overlap).
- JSC (IoU): Calculates the ratio of intersection to union between predicted and ground truth regions, ranging from 0 (no overlap) to 1 (complete overlap).
- HD: Quantifies the maximum spatial deviation between predicted and ground truth regions, ranging from 0 (perfect alignment) to ∞ (maximum discrepancy).
- Precision: The proportion of true positives out of all positive predictions, ranging from 0 to 1, with 1 indicating no false positives.
- Recall: The ability to correctly identify all relevant positive regions, ranging from 0 (no recall) to 1 (perfect recall).
- Specificity: The ability to correctly identify all relevant negative regions (true negative rate), ranging from 0 to 1.

A comparison of the average DSC, JSC, HD, precision, recall, and specificity values across the 20 different cases of defected skulls (covering four defect types and five regions) chosen from the testing dataset and reconstructed by CRIGNet is presented in Table 2. The high values for DSC, JSC, HD, precision, recall, and specificity, and low values for HD, as shown in Table 2, indicate a nearly perfect reconstruction, thereby proving the effectiveness of the model in cranial reconstruction across diverse sets of shapes and regions.

Table 2 presents the average values of the performance metrics of CRIGNet for each defect type, with the highest values in each parameter emboldened for clarity. It can be seen from Table 2 that Case 11 (Triangular Front) demonstrated the lowest HD (3.8103) and highest recall (0.9994) and specificity (0.9998), while Case 14 (Triangular Top) achieved the highest DSC (0.9982), JSC (0.9964), and precision (0.9971), and was tied with Case 11 (Triangular Front) for the highest specificity (0.9998).

### 3.5. Comparative Analysis of CRIGNet and Other AI Models

Several state-of-the-art models were trained and tested on the same dataset, with six yielding satisfactory results. Brief details of these models are as follows:

1. Autoencoder [11]*: A compact encoder–decoder architecture using 3D convolutions and Max-Pooling, ending with a Sigmoid activation for voxel intensity normalisation.
2. DCGAN [44]*: Incorporates 3D convolutional blocks with Leaky ReLU for feature extraction, a modified discriminator with 64 initial channels, and a Sigmoid-activated decoder.
3. SegNet [28]*:A 3D adaptation of SegNet, replacing 2D operations with 3D convolutions and Max-Unpooling, ending with Sigmoid activation for binary outputs.
4. 3DCNN [6]*: A simplified architecture with two 3D convolutional layers in both the encoder and decoder, concluding with Sigmoid activation.
5. DeepMedic [29]*: Focused on high-resolution pathways without residual connections, using 1 × 1 × 1 convolutions and Sigmoid activation for binary classification.
6. Ensemble: Combines predictions from CRIGNet, SegNet, DeepMedic, and 3DCNN through a meta model with 3D convolution and Sigmoid activation for final output.

Table 3 presents a comparative analysis of autoencoder*, DCGAN*, SegNet*, 3DCNN*, DeepMedic*, ensemble, and CRIGNet based on various performance metrics averaged over a testing dataset of 720 files. It can be observed from the table that CRIGNet excels with the highest DSC (0.9948), JSC (0.9897), precision (0.9920), recall (0.9977), and specificity (0.9994), alongside a low HD value (4.6317 mm). The ensemble model, as a strong alternative, achieved a DSC of 0.9844, JSC of 0.9693, precision of 0.9774, recall of 0.9915, specificity of 0.9984, and HD of 4.1297 mm. Despite the impressive performance of the Ensemble model, CRIGNet outperforms it across all key metrics.

CRIGNet’s high DSC, JSC, and low HD indicate exceptional alignment between the reconstructed and actual skulls, reflecting its high accuracy in capturing both detailed structures and overall shapes. Its high precision demonstrates its effectiveness in minimizing false positives, while the high recall underscores its ability to identify true positives and minimise false negatives. The excellent specificity value further highlights its robustness in avoiding false positives. Although the ensemble model shows competitive performance with a slightly lower HD value (4.1297 mm), CRIGNet’s superior results across other metrics affirm its greater accuracy and efficiency for cranial reconstruction.

**Figure 7:**
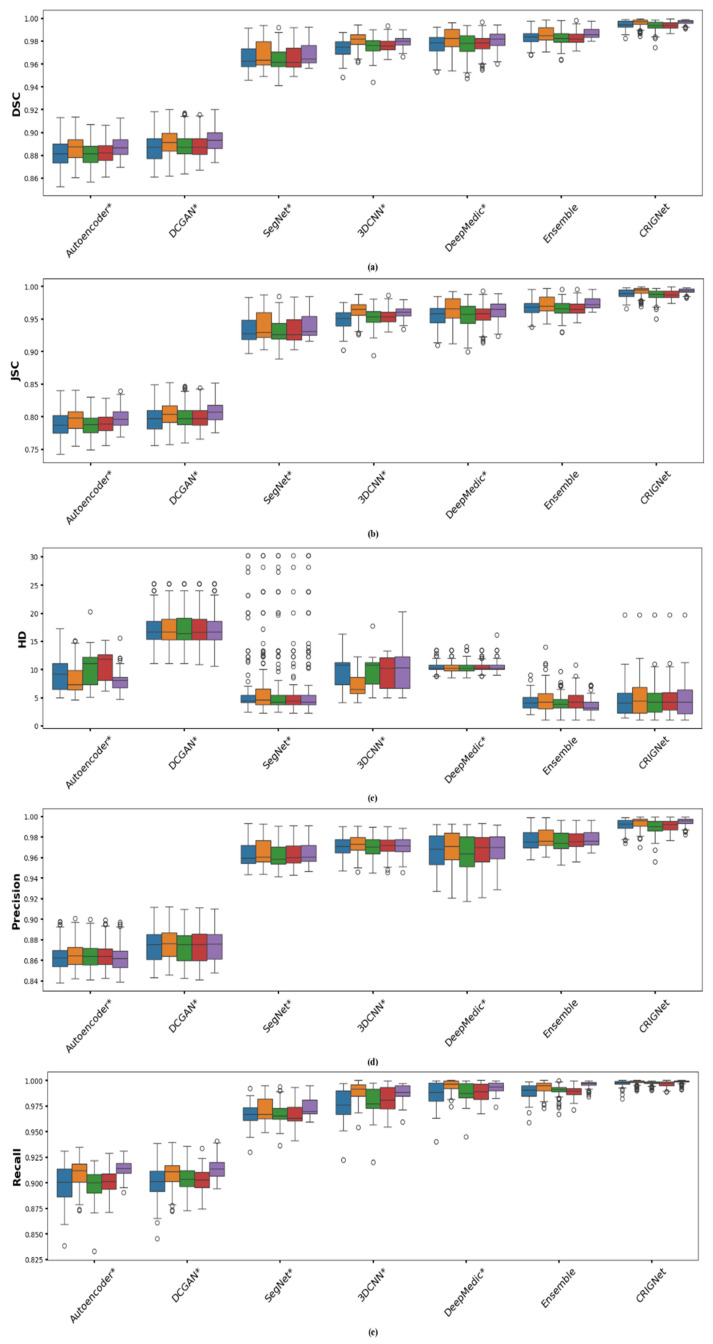

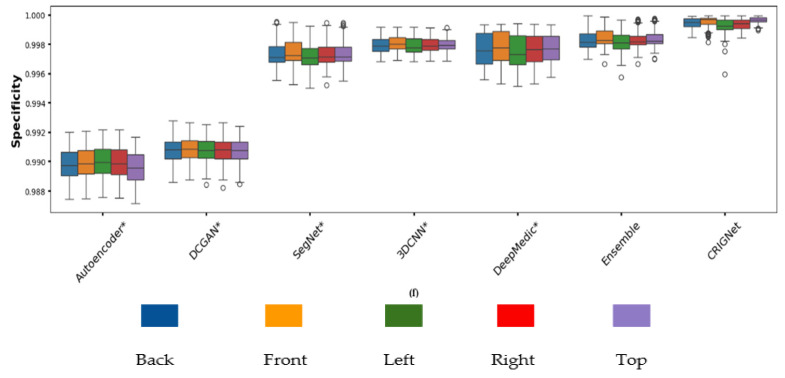
Box plots for quantitative comparison of CRIGNet with existing AI models for cranial reconstruction based on (**a**) DSC, (**b**) JSC, (**c**) HD, (**d**) precision, (**e**) recall, and (**f**) specificity. * Represents the modified architecture as described in Section 3.5.

Figure 7 shows the box plots of the region-wise performance for cranial reconstruction across all AI models under comparison, as listed in Section 3.5. The reliability of CRIGNet is evident from its superior DSC values in Figure 7a, characterized by the smallest box height and whisker length, alongside the largest median values. Figure 7b further reinforces the consistency of CRIGNet in terms of the more stringent JSC measure, visually presenting the smallest Interquartile Range (IQR) of its result metrics. For HD values, although the IQR of the CRIGNet-based reconstruction is slightly larger than that of the ensemble, SegNet, and DeepMedic models, as shown in Figure 7c, it is compensated by lower median values. This increase in box size can be attributed to the nature of the HD calculation, which is sensitive to the presence of outlying pixels in the model’s prediction. However, this does not pose a threat to CRIGNet’s dependability, as these noisy pixels can be easily removed through denoising of the output. The highest values and lower variability in precision, as presented in Figure 7d, indicate the minimal generation of false positives (pepper noise) by CRIGNet. The recall and specificity values, shown in Figure 7e,f, highlight CRIGNet’s unmatched performance, with extremely narrow dispersion, minimal outlying values, and symmetry in the metric values.

Overall, CRIGNet demonstrates uniformity in region-wise performance, as opposed to other models, showing generally lower median values in the back region. It has been observed that reconstructive performance is higher in the top region for all models in terms of DSC and JSC values, though CRIGNet maintains a leading edge. In general, the lower variability in CRIGNet’s performance across all test cases and metrics is a testament to its supremacy.

### 3.6. Geometrical Accuracy and Load Testing of PEEK Based Implants for Validation

#### 3.6.1. CAD-Generated Implant

**Figure 8:**
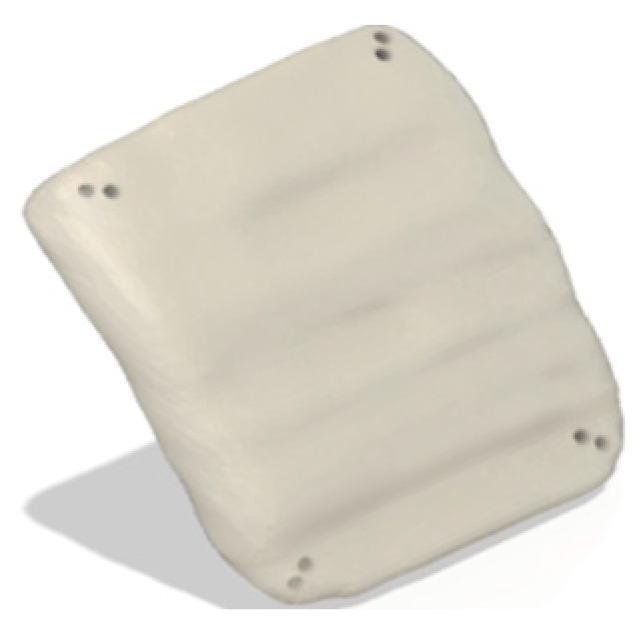
CAD-generated implant (Cubical Left).

**Figure 9:**
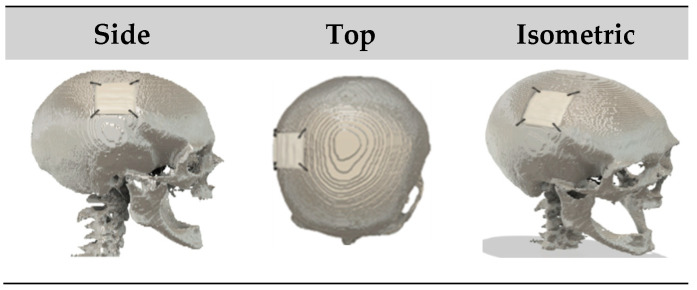
Different views of the skull (grey) with CAD-generated implant (white) and four linear fixture plates.

**Figure 10:**
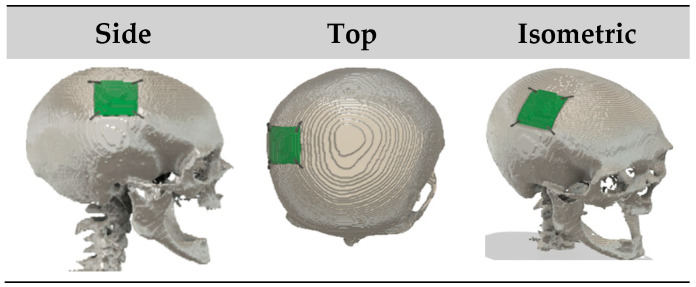
Different views of the skull (grey) with CRIGNet-generated implant (green) and four linear fixture plates.

A 3D model of a defected skull from the testing dataset was reconstructed using the interpolation technique in 3D Slicer (v5.0.2, The Slicer community) software [30,45]. There, a 3D model of the implant is created using CAD modelling, as shown in Figure 8. The design of the skull-implant fixture plate assemblies created using AUTODESK Fusion 360 is illustrated in Figure 9 and Figure 10, which show the assemblies with the CAD-generated and CRIGNet-generated implants, respectively. Both assemblies used a symmetrical arrangement of four linear fixture plates at key points, with pins inserted on each side of the implant and the skull surface to enhance symmetry and minimise stress concentration [23].

#### 3.6.2. Geometrical Accuracy

**Figure 11:**
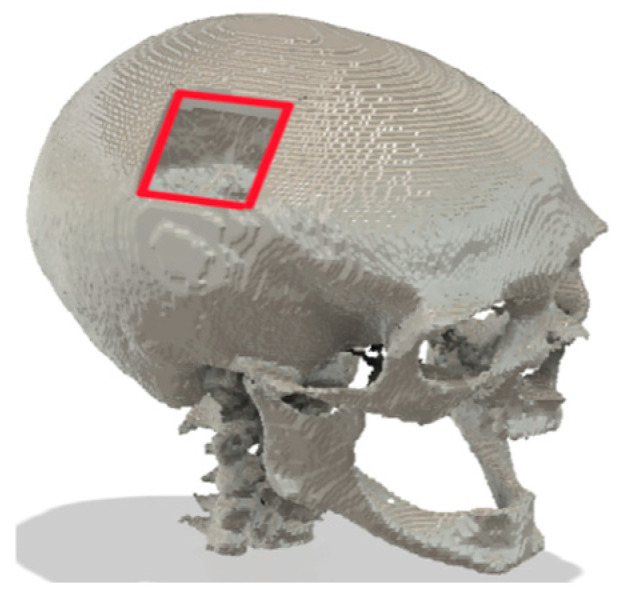
Cavity (red bound) of the defected skull.

This section compares the edge gaps between the boundaries of CAD-generated implants and the cavity boundaries. Similarly, the edge gaps between the boundaries of AI-generated CRIGNet implants and the cavity boundaries are also compared to assess the best fitment amongst both implants. The cavity boundaries, which are highlighted in red in Figure 11, serve as a reference for this comparison.

#### 3.6.3. Implant Edge Gap Measurement

**Figure 12:**
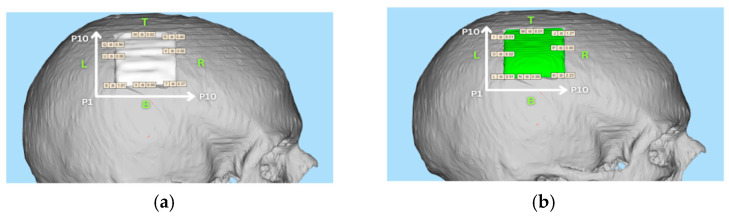
Edge gap between the boundaries of cavity of defected skull for (**a**) CAD-generated implant and (**b**) CRIGNet-generated implant.

Measurements were taken at ten distinct locations (P1 to P10) on each side of the cavity, labelled as left (L), bottom (B), right (R), and top (T), as shown in Figure 12a,b, which illustrate the edge gaps for the CAD-generated and CRIGNet-generated implants, respectively.

The average edge gap, in mm, for each side (L, B, R, and T) as labelled in Figure 12, has been provided in Table 4, along with the overall average edge gap, in mm, of the CAD-generated and CRIGNet-generated implants.

As shown in Table 4, the average edge gap between the CAD-generated implant and the cavity of the defected skull was 0.18 mm across all sides. In comparison, the CRIGNet-generated implant exhibited an average gap of 0.26 mm, which is comparable, suggesting that the CRIGNet-generated implant can be satisfactorily fitted within the cavity.

#### 3.6.4. Load Testing Through Simulation

An ANSYS 15 2022 R2 FEA simulation was conducted on the skull-implant four-fixture-plate assembly to evaluate its overall deformation and von Mises stress under direct top-down loading. The material of the implant was PEEK. The skull model was first idealised using CAD tools for repair and mending, then transformed into a solid body suitable for export in a Standard for the Exchange of Product Data (STEP) format. A tetrahedral mesh was generated using an element size of 1 mm and a quadratic element order, refined with span angles ranging from 12° to 36° and a high smoothing factor, ensuring 0% defects [46]. The simulation incorporated multiple boundary conditions, including external load, intracranial pressure (ICP), and immobilisation support. A fixed plane at the skull base was used to simulate immobilisation. A distributed external load of 1780 N was applied to replicate impact pressures experienced during trauma scenarios like road accidents or free falls [47,48]. The ICP, which typically ranges from 7 to 15 mmHg in adults, was set at the upper physiological limit of 15 mmHg to simulate post-surgical conditions and normal variations due to body posture [18,19]. The assembly base was provided with permanent support, and the static structural analysis was performed using the ANSYS Parametric Design Language (APDL).

The material properties assigned to each component of the assembly are summarised in Table 5. The implant was made from PEEK, chosen for its favourable mechanical and biocompatible properties, while genuine human bone was modelled for the defected skull. Fixture plates and pins, considered the most load-prone components, were made of titanium alloy (Ti-6Al-4V) [49].

**Figure 13:**
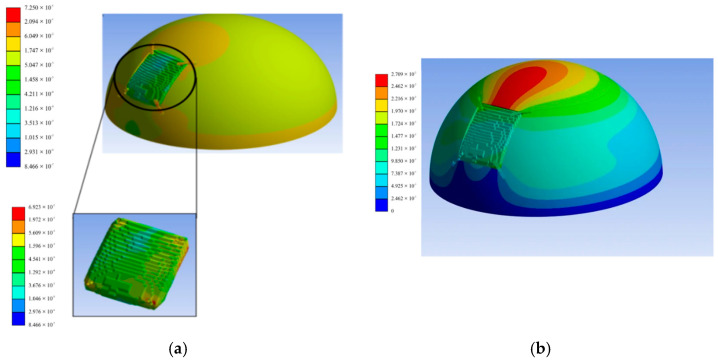
Simulation of CRIGNet-generated implant in ANSYS: (**a**) equivalent von Mises stress distribution and (**b**) total deformation.

The FEA results, as shown in Figure 13a,b, demonstrate that the CRIGNet-generated PEEK-based implant exhibited von Mises stresses of 0.72 MPa, well within the yielding limits to avoid failure under heavy external loading, and a minimal deformation of 0.03 mm, respectively. With evenly distributed fixtures, the stress range of 0.0166–0.0174 MPa was achieved, reducing implant stress by transferring loads to the larger surface area of the titanium plates. These results highlight PEEK’s superior stress-relieving properties compared to titanium, facilitated by its lower elastic modulus and optimised fixation design. As shown in Figure 13a, von Mises stresses within the assembly remained well within yielding limits, confirming the implant’s safety and load-sustaining capacity [51]. PEEK’s porous structure further enhances load-sharing, outperforming titanium in stress mitigation [52]. Its biocompatibility and 3D printability make it an ideal alternative to Ti-6Al-4V for cranial implants, providing reliable, high-performance solutions for cranial reconstruction.

#### 3.6.5. Time Efficiency

The proposed AI-based implant generation framework significantly outperformed state-of-the-art CAD methods in terms of time efficiency. Generating a CAD implant involves a series of time-consuming steps: loading the DICOM file into 3D Slicer (approximately 10 s), visualising the defect (5 s), reconstructing the implant using CAD techniques (20 min for mirroring and 30 min for interpolation), post-processing the defect (10 min), and exporting the STL file (5 s). This process takes a total of 40–45 min.

In contrast, the CRIGNet framework processes raw input data and generates a well-fitting implant in just 25–30 s. This includes denoising (approximately 3 s), CCA (around 17 s), resampling (less than a second), reconstruction (about 2 s), resolution enhancement (approximately 0.1 s), and implant generation (about 2 s).

This remarkable efficiency—80 times faster than CAD-based methods—is particularly advantageous in time-sensitive scenarios where traditional CAD modelling might be impractical. It significantly reduces the risk for critical patients by enabling quicker surgical interventions.

## 4. Discussion

Cranioplasty is a critical surgical procedure. For a long time, CAD-generated implants have dominated the industry. However, traditional cranial defect repair methods are often time-consuming and labour-intensive, adding complexity to surgical procedures. With advancements in technology, AI-based alternatives have emerged as viable solutions for this crucial process of cranial reconstruction. Despite these innovations, it is essential to ensure that the generated implants meet the high standards of accuracy and precision required for medical applications, address varying patient needs and defect types, and minimise implant generation time. Therefore, these implants must perform exceptionally well across evaluation metrics such as DSC, JSC, precision, and recall. Additionally, comparing these AI-generated implants with those created using the CAD method—where research remains limited—is necessary to validate their efficiency and efficacy.

This research addressed these challenges by proposing an integrated solution, CRIGNet, that provides a highly accurate reconstruction and implant generation while accommodating the versatility of bone defects and the need for high-speed delivery. It includes a robust preprocessing pipeline capable of handling variations in input data, ensuring consistency and quality. This pipeline incorporates techniques such as thresholding, denoising, CCA, and image alignment to mitigate inaccuracies in defect segmentation [6,8]. A key component is the defect generation process, which produces defects of various shapes across multiple skull regions. Unlike traditional symmetry-based models [1,7], CRIGNet employed extensive augmentation techniques to expand the dataset from 396 healthy skulls extracted from the MUG500+ dataset to 2160 data pairs.

During training, the AI model diagnosed various defect regions and shapes, dynamically adjusting its weights and biases based on emerging patterns. The model’s exceptional results can be attributed to carefully designed architectural features, including residual connections that mitigate the vanishing gradient problem while preserving both macro- and micro-level morphological details. The incorporation of batch normalisation and the ReLU activation function stabilized the learning process for complex, non-linear defect patterns. Furthermore, the resolution enhancement network significantly boosted performance across multiple evaluation metrics. CRIGNet demonstrated outstanding performance, achieving an average DSC of 0.9948, JSC of 0.9897, precision of 0.9920, recall of 0.9977, specificity of 0.9994, and an HD value of 4.6317 mm. The box plot analysis highlighted CRIGNet’s superior accuracy and reduced variability in reconstruction performance compared to state-of-the-art methods [6,11,28,29,30]. Given the critical importance of accuracy and precision in medical applications, these results underscore CRIGNet’s reliability and its ability to generate implants precisely tailored to individual patient needs, addressing key clinical requirements.

Material selection plays a pivotal role in ensuring the sufficient mechanical strength, safety, and effectiveness of cranial implants. CRIGNet-generated implants utilize PEEK, which offers superior stress-relieving properties, durability, and biocompatibility compared to the widely used titanium material. PEEK’s natural radiolucency ensures compatibility with X-ray, CT, ultrasonic, and MRI imaging, allowing artefact-free follow-up treatments [20,21,22]. It also supports bone growth, making it particularly suitable for adolescents and children. Metal implants often cause discomfort to patients during winter due to their higher thermal conductivity. Titanium’s thermal conductivity of nearly 21.9 W/mK is significantly higher than PEEK’s thermal conductivity, which is only 0.29 W/mK. This enhances its prospects as an implant material [53,54]. Additionally, PEEK demonstrates low plaque affinity, chemical resilience, and excellent aesthetic and functional outcomes in cranioplasty. However, PEEK’s applications remain constrained due to its higher costs—nearly twice that of titanium implants—which influence its demand and availability [55]. These factors lead surgeons and patients to prefer titanium-based implants for emergency and routine cranial reconstruction. However, for adolescents and children, where bone growth is significant, and in cases with higher risks of implant rejection, PEEK-based implants are recommended. Bone grafts remain the preferred choice for small defects or cases prioritizing natural integration. FEA simulations validated CRIGNet-generated PEEK implants, demonstrating that von Mises stresses (0.72 MPa) and deformations (0.03 mm) remain within safe thresholds, ensuring long-term patient safety and reliability [51].

Efficiency in implant generation is another key benefit of CRIGNet. The AI framework generates implants in 25–30 s—a significant improvement over the 40–45 min required for manual CAD-based workflows. This 98.75% reduction in time minimises the surgeon’s effort and eliminates the need for skilled manpower, which is typically required for manual generation, making CRIGNet a scalable and sustainable alternative for cranial reconstruction.

Despite its remarkable performance, CRIGNet has scope for further improvement. The model’s ability to handle complex cranial defect reconstructions remains underexplored due to the limited availability of training datasets for such cases. Consequently, manual intervention and CAD-based methods are still necessary in these scenarios. Additionally, a comparison of edge gaps between CRIGNet-generated implants (0.26 mm) and CAD-generated implants (0.18 mm) reveals slight differences. Although acceptable, these highlight potential for improved precision. Optimising the preprocessing pipeline with advanced cropping techniques and automating case-specific thresholding could further refine performance. Lastly, thorough clinical validation is essential to ensure consistent real-world outcomes.

Nevertheless, CRIGNet provides significant advantages over traditional CAD methods and other AI solutions, offering improved geometrical accuracy, mechanical strength, faster design, and processing capabilities.

## 5. Conclusions and Future Scope

This study proposed an advanced and novel AI-based integrated solution for cranial reconstruction and implant generation, called CRIGNet, representing a significant advancement in the application of AI in cranioplasty. By integrating a comprehensive preprocessing pipeline and developing a method for generating a wide range of cranial defects across distinct skull regions, CRIGNet diagnoses defect patterns and delivers precise, patient-specific implants. CRIGNet outperforms existing AI solutions in terms of DSC, JSC, and HD scores, and surpasses traditional CAD-based methods in geometrical accuracy, mechanical strength, and time efficiency. Notably, CRIGNet achieves this in a significantly reduced time duration of 25–30 s, compared to the 40–45 min taken by CAD-based methods, which significantly reduces patient wait times and enables timely medical care. Additionally, the creation of a dataset with 2160 skulls featuring defects of various shapes and locations serves as a valuable digital reference for future studies and research in this domain.

The social and clinical impacts of this AI framework are profound. By reducing reliance on specialized human resources and automating the implant generation process, CRIGNet makes personalized cranial implants more accessible, particularly in regions with limited healthcare infrastructure. These implants can be pre-planned and directly delivered to the surgical site, eliminating the need for frequent patient visits before surgery. Future research will focus on training the model on defect shapes that more closely resemble real-life cases, with rigorous clinical validation. Along with dataset expansion, further improvements in model architecture and preprocessing pipelines will be pursued to enhance the accuracy of implant fitment.

## Figures and Tables

**Table 1:** Comparative analysis of existing cranial reconstruction techniques.

Year	Author	Dataset	Methodology	Merits	Demerits
2020	Kodym et al. [9]	CQ500 dataset, SkullBreak dataset	3D CNN cascade with symmetrised low-resolution input, generative model	High resolution, clinically viable	Cross assessment may not be accurate
2020	Matzkin et al. [13]	CQ500 public database; training images: 100, test images: 110	DE-UNet and DE-Shape-UNet models, incorporating shape priors for improved robustness	Better accuracy for in-distribution defects, improved robustness for out-of-distribution cases	Lower overall accuracy compared to DE-UNet
2021	Li et al. [6]	167 healthy skulls segmented from head CT scans, additional data from craniotomy case	Data-driven 3D shape completion encoder–decoder and 3D CNN	Accurate fit in software, ability to generate multiple data pairs from one skull	Manual work is required for a 3D-printed implant
2021	Yang et al. [15]	AutoImplant 2021 challenge: 570 training cases, 100 evaluation samples	Ensemble of slice-based networks with CNN and RNN models	Improved performance with ensemble strategy and impurity filters	Isolated noise in predictions
2022	Wodzinski et al. [10]	AutoImplant 2021 challenge datasets: SkullBreak (Task 1), real cranial defects (Task 2), and SkullFix (Task 3)	U-Net with residual blocks	Modelled fast, fully automatic, personalised cranial implants	Quantitative results varied with defect size and position; fronto-orbital defects had lower scores
2022	Wu et al. [7]	DICOM metadata, Taiwan	3D deep learning network based on an enhanced three-dimensional autoencoder	Little or no post-processing required, computationally efficient	Limited to the upper part of the cranium, post-processing required for clinical practice
2023	Li et al. [11]	MUG500+, SkullFix	Autoencoder model based on MONAI framework	Open source, easy integration with MONAI, efficient processing using GPUs	Not enough information about specific parameters provided
2024	Resmi et al. [16]	2020 Auto Implant challenge	Skull reconstruction using integration of 3D U-Net with Transformers	Novel method addressed the limitations of traditional CAD techniques	Lower resolution images affected the generalizability of the results to high-resolution data
2024	Li et al. [17]	AutoImplant II challenge, MUG500+ dataset	Statistical Shape Model (SSM), Principal Component Analysis	Robust against large and complex defects, mathematically explicit shape representation	Suboptimal performance on synthetic defects compared to CNN-based approaches
2024	Wodzinski et al. [12]	SkullFix, SkullBreak	Deep deformable masked autoencoder	Efficient reconstruction in real time	No testing performed

**Table 2:** Performance of CRIGNet in cranial reconstruction for various defect cases.

Case No.	Case	DSC	JSC	HD	Precision	Recall	Specificity
1	Cylindrical Front	0.9964	0.9928	4.5240	0.9942	0.9986	0.9996
2	Cylindrical Left	0.9895	0.9792	4.6443	0.9825	0.9966	0.9987
3	Cylindrical Back	0.9922	0.9845	4.3930	0.9893	0.9951	0.9992
4	Cylindrical Top	0.9961	0.9921	5.0810	0.9944	0.9978	0.9996
5	Cylindrical Right	0.9926	0.9854	4.4012	0.9920	0.9933	0.9994
6	Spherical Front	0.9919	0.9840	5.5218	0.9874	0.9966	0.9991
7	Spherical Left	0.9933	0.9867	4.7579	0.9890	0.9976	0.9992
8	Spherical Back	0.9953	0.9906	4.0946	0.9925	0.9980	0.9995
9	Spherical Top	0.9966	0.9933	4.3680	0.9951	0.9982	0.9997
10	Spherical Right	0.9928	0.9857	4.3359	0.9886	0.9971	0.9992
11	Triangular Front	0.9980	0.9959	3.8103	0.9965	0.9994	0.9998
12	Triangular Left	0.9973	0.9947	3.8993	0.9964	0.9983	0.9997
13	Triangular Back	0.9977	0.9954	3.9395	0.9963	0.9991	0.9997
14	Triangular Top	0.9982	0.9964	4.0790	0.9971	0.9993	0.9998
15	Triangular Right	0.9972	0.9944	4.1570	0.9958	0.9986	0.9997
16	Cubical Front	0.9973	0.9946	5.3278	0.9953	0.9993	0.9997
17	Cubical Left	0.9934	0.9869	5.1989	0.9890	0.9979	0.9992
18	Cubical Back	0.9925	0.9851	5.4119	0.9882	0.9968	0.9992
19	Cubical Top	0.9955	0.9910	5.2140	0.9919	0.9991	0.9994
20	Cubical Right	0.9917	0.9836	5.4810	0.9867	0.9969	0.9991

**Table 3:** Comparison of CRIGNet with other AI models ** for cranial reconstruction.

Model	DSC	JSC	HD	Precision	Recall	Specificity
Autoencoder *	0.8836	0.7917	9.2881	0.8640	0.9043	0.9898
DCGAN *	0.8899	0.8018	17.276	0.8748	0.9057	0.9908
SegNet *	0.9669	0.9361	6.3500	0.9643	0.9695	0.9974
3DCNN *	0.9772	0.9555	9.2135	0.9713	0.9833	0.9980
Deep Medic *	0.9786	0.9582	10.404	0.9671	0.9905	0.9976
Ensemble	0.9844	0.9693	4.1297	0.9774	0.9915	0.9984
CRIGNet	0.9948	0.9897	4.6317	0.9920	0.9977	0.9994

* Represents the modified architecture as described above. ** The order of models is according to the increasing order of their DSC.

**Table 4:** Average edge gap between the edges of the CRIGNet-generated implant and the CAD-generated implant with the edges of the cavity within the defected skull.

Cranial Implants/Side Measurement	L	R	T	B	Overall Average Gap
CAD-generated	0.97	0.09	0.17	0.15	0.18
CRIGNet-generated	0.15	0.35	0.23	0.33	0.26

**Table 5:** Properties of materials used in the assembly [18,50].

Material	Density (kg/m^3^)	Elastic Modulus (MPa)	Poisson’s Ratio	Yield Strength (MPa)
Autologous Bone (Defected Skull)	4430	15,000	0.30	133
PEEK (CRIGNet-Generated Implant)	1240	4000	0.44	100
Ti-6Al-4V (Fixture and Pin)	4500	110,000	0.30	800

## Data Availability

Data can be shared with the reader on request.

## Abbreviations

The following abbreviations are used in this manuscript:

AI	Artificial Intelligence
APDL	ANSYS Parametric Design Language
Back	Occipital
CAD	Computer–Aided Design
CCA	Connected Component Analysis
CNN	Convolutional Neural Network
CRIGNet	Cranial Reconstruction and Implant Generation Network
CT	Computed Tomography
DICOM	Digital Imaging and Communications in Medicine
DSC	Dice Similarity Coefficient
FEA	Finite Element Analysis
Front	Frontal
GANs	Generative Adversarial Networks
HD	Hausdorff distance
IoU	Intersection to Union
IQR	Interquartile Range
JSC	Jaccard Similarity Coefficient
Left	Left Parietal Temporal
MONAI	Medical Open Network for Artificial Intelligence
MRI	Magnetic resonance imaging
NRRD	Nearly Raw Raster Data
PCA	Principal Component Analysis
PEEK	Polyether-Ether-Ketone
Right	Right Parietal Temporal
RNN	Recurrent Neural Network
SSM	Statistical Shape Models
STEP	Standard for the Exchange of Product Data
Top	Parietal

## References

[1] Lee M.-Y., Chang C.-C., Lin C.-C., Lo L.-J., Chen Y.-R. (2002). Custom implant design for patients with cranial defects. IEEE Eng. Med. Biol. Mag..

[2] Jardini A.L., Larosa M.A., Maciel Filho R., Zavaglia C.A., Bernardes L.F., Lambert C.S., Calderoni D.R., Kharmandayan P. (2014). Cranial reconstruction: 3D biomodel and custom-built implant created using additive manufacturing. J. Craniomaxillofac. Surg..

[3] Oh J.H. (2018). Recent advances in the reconstruction of cranio-maxillofacial defects using computer-aided design/computer-aided manufacturing. Maxillofac. Plast. Reconstr. Surg..

[4] van de Belt H., Neut D., Schenk W., van Horn J.R., van der Mei H.C., Busscher H.J. (2001). Infection of orthopaedic implants and the use of antibiotic-loaded bone cements: A review. Acta Orthop. Scand..

[5] Rudman K., Hoekzema C., Rhee J. (2011). Computer-assisted innovations in craniofacial surgery. Facial Plast. Surg..

[6] Li J., von Campe G., Pepe A., Gsaxner C., Wang E., Chen X., Zefferer U., Tödtling M., Krall M., Deutschmann H. (2021). Automatic skull defect restoration and cranial implant generation for cranioplasty. Med. Image Anal..

[7] Wu C.-T., Yang Y.-H., Chang Y.-Z. (2022). Three-dimensional deep learning to automatically generate cranial implant geometry. Sci. Rep..

[8] Li J., Pepe A., Schwarz-Gsaxner C., Egger J. (2021). An Online Platform for Automatic Skull Defect Restoration and Cranial Implant Design. Proc. SPIE.

[9] Kodym O., Španěl M., Herout A. (2020). Skull shape reconstruction using cascaded convolutional networks. Comput. Biol. Med..

[10] Wodzinski M., Daniol M., Socha M., Hemmerling D., Stanuch M., Skalski A. (2022). Deep learning-based framework for automatic cranial defect reconstruction and implant modelling. Comput. Methods Programs Biomed..

[11] Li J., Ferreira A., Puladi B., Alves V., Kamp M., Kim M., Nensa F., Kleesiek J., Ahmadi S.-A., Egger J. (2023). Open-source skull reconstruction with MONAI. Softw. X.

[12] Wodzinski M., Hemmerling D., Daniol M. (2024). Automatic Cranial Defect Reconstruction with Self-Supervised Deep Deformable Masked Autoencoders. arXiv.

[13] Matzkin F., Newcombe V., Glocker B., Ferrante E. (2020). Cranial Implant Design via Virtual Craniectomy with Shape Priors. Lecture Notes in Computer Science (Including Subseries Lecture Notes in Artificial Intelligence and Lecture Notes in Bioinformatics.

[14] Kwarciak K., Wodziński M. Deep Generative Networks for Heterogeneous Augmentation of Cranial Defects. Proceedings of the 2023 IEEE/CVF International Conference on Computer Vision Workshops (ICCVW).

[15] Yang B., Fang K., Li X. (2021). Cranial implant prediction by learning an ensemble of slice-based skull completion networks. Lecture Notes in Computer Science.

[16] Resmi S., Singh R.P., Palaniappan K. (2024). Automatic skull shape completion of defective skulls using transformers for cranial implant design. Procedia Comput. Sci..

[17] Li J., Ellis D.G., Pepe A., Gsaxner C., Aizenberg M.R., Kleesiek J., Egger J. (2024). Back to the roots: Reconstructing large and complex cranial defects using an image-based statistical shape model. J. Med. Syst..

[18] Bogu V.P., Kumar Y.R., Khanara A.K. (2017). Modelling and structural analysis of skull/cranial implant: Beyond midline deformities. Acta Bioeng. Biomech..

[19] Jindal P., Chaitanya, Bharadwaja S.S.S., Rattra S., Pareek D., Gupta V., Breedon P., Reinwald Y., Juneja M. (2023). Optimising Cranial Implant and Fixture Design Using Different Materials in Cranioplasty. Proc. Inst. Mech. Eng. Part L J. Mater. Des. Appl..

[20] Mallya P.K., Juneja M. (2021). Rapid Prototyping of Orthopaedic Implant Materials for Cranio-Facial Reconstruction: A Survey. Mater. Today Proc..

[21] Ma H., Suonan A., Zhou J., Yuan Q., Liu L., Zhao X., Lou X., Yang C., Li D., Zhang Y.-G. (2021). PEEK (Polyether-Ether Ketone) and Its Composite Materials in Orthopaedic Implantation. Arab. J. Chem..

[22] Zhang J., Tian W., Chen J., Yu J., Zhang J., Chen J. (2019). The Application of Polyetheretherketone (PEEK) Implants in Cranioplasty. Brain Res. Bull..

[23] Jindal P., Bharadwaja S.S.S., Rattra S., Chaitanya, Gupta V., Breedon P., Reinwald Y., Juneja M. (2023). Designing cranial fixture shapes and topologies for optimising PEEK implant thickness in cranioplasty. Proc. Inst. Mech. Eng. Part L J. Mater. Des. Appl..

[24] Haleem A., Javaid M. (2019). Polyether Ether Ketone (PEEK) and Its 3D Printed Implants Applications in the Medical Field: An Overview. Clin. Epidemiol. Global Health.

[25] Verma S., Sharma N., Kango S., Sharma S. (2021). Developments of PEEK (Polyetheretherketone) as a Biomedical Material: A Focused Review. Eur. Polym. J..

[26] de Ruiter L., Janssen D., Briscoe A., Verdonschot N. (2017). The Mechanical Response of a Polyetheretherketone Femoral Knee Implant under a Deep Squatting Loading Condition. Proc. Inst. Mech. Eng. Part H.

[27] Mian S.H., Moiduddin K., Elseufy S.M., Alkhalefah H. (2022). Adaptive Mechanism for Designing a Personalized Cranial Implant and Its 3D Printing Using PEEK. Polymers.

[28] Badrinarayanan V., Kendall A., Cipolla R. (2015). SegNet: A Deep Convolutional Encoder-Decoder Architecture for Image Segmentation. arXiv.

[29] Kamnitsas K., Ledig C., Newcombe V.F.J., Simpson J.P., Kane A.D., Menon D.K., Rueckert D., Glocker B. (2017). Efficient multi-scale 3D CNN with fully connected CRF for accurate brain lesion segmentation. Med. Image Anal..

[30] Fedorov A., Beichel R., Kalpathy-Cramer J., Finet J., Fillion-Robin J.-C., Pujol S., Bauer C., Jennings D., Fennessy F.M., Sonka M. (2012). 3D Slicer as an Image Computing Platform for the Quantitative Imaging Network. Magn. Reson. Imaging.

[31] Li J., Krall M., Trummer F., Memon A.R., Pepe A., Gsaxner C., Jin Y., Chen X., Deutschmann H., Zefferer U. (2021). MUG500+: Database of 500 high-resolution healthy human skulls and 29 craniotomy skulls and implants. Data Brief.

[32] Kaur R., Juneja M., Mandal A.K. (2018). A comprehensive review of denoising techniques for abdominal CT images. Multimedia Tools Appl..

[33] Juneja M., Saini S.K., Kaul S., Acharjee R., Thakur N., Jindal P. (2021). Denoising of magnetic resonance imaging using Bayes shrinkage-based fused wavelet transform and autoencoder-based deep learning approach. Biomed. Signal Process. Control.

[34] Majanga V., Viriri S. (2021). Dental images’ segmentation using threshold connected component analysis. Comput. Intell. Neurosci..

[35] Wu C.-T., Yang Y.-H., Chang Y.-Z. (2023). Creating high-resolution 3D cranial implant geometry using deep learning techniques. Front. Bioeng. Biotechnol..

[36] Li J., Gsaxner C., Pepe A., Morais A., Alves V., von Campe G., Wallner J., Egger J. (2021). Synthetic skull bone defects for automatic patient-specific craniofacial implant design. Sci. Data.

[37] Diakogiannis F.I., Waldner F., Caccetta P., Wu C. (2020). ResUNet-a: A deep learning framework for semantic segmentation of remotely sensed data. ISPRS J. Photogramm. Remote Sens..

[38] Bjorck N., Gomes C.P., Selman B., Weinberger K.Q., Bengio S., Wallach H., Larochelle H., Grauman K., Cesa-Bianchi N., Garnett R. (2018). Understanding Batch Normalisation. Advances in Neural Information Processing Systems.

[39] Agarap A.F. (2018). Deep Learning Using Rectified Linear Units (ReLU). arXiv.

[40] Sharma S., Sharma S., Athaiya A. (2017). Activation Functions in Neural Networks. Int. J. Eng. Appl. Sci. Technol..

[41] Vani S., Rao T.V.M. An experimental approach towards the performance assessment of various optimizers on convolutional neural network. Proceedings of the 2019 3rd International Conference on Trends in Electronics and Informatics (ICOEI).

[42] Huttenlocher D.P., Klanderman G.A., Rucklidge W.J. (1993). Comparing images using the Hausdorff distance. IEEE Trans. Pattern Anal. Mach. Intell..

[43] Juneja M., Thakur N., Thakur S., Uniyal A., Wani A., Jindal P. (2020). GC-NET for classification of glaucoma in the retinal fundus image. Mach. Vis. Appl..

[44] Ahmadian H., Mageswaran P., Walter B.A., Blakaj D.M., Bourekas E.C., Mendel E., Marras W.S., Soghrati S. (2022). Toward an artificial intelligence-assisted framework for reconstructing the digital twin of vertebrae and predicting its fracture response. Int. J. Numer. Methods Biomed. Eng..

[45] 3D Slicer Image Computing Platform. https://www.slicer.org/.

[46] Kung W.-M., Tzeng I.-S., Lin M.-S. (2020). Three-dimensional CAD in skull reconstruction: A narrative review with focus on cranioplasty and its potential relevance to brain sciences. Appl. Sci..

[47] Morselli C., Zaed I., Tropeano M.P., Cataletti G., Iaccarino C., Rossini Z., Servadei F. (2019). Comparison between the Different Types of Heterologous Materials Used in Cranioplasty: A Systematic Review of the Literature. J. Neurosurg. Sci..

[48] Ganatsios S., Maropoulos S., Tsouknidas A., Papanikolaou S. (2011). Porosity-Mechanical Behaviour Correlation in Cranial Implants. Romanian Rev. Precision Mech..

[49] Juneja M., Bajaj D., Thakur N., Jindal P. (2024). Reproduction of human dental models using different 3D printing techniques. Proc. Inst. Mech. Eng. Part E J. Process Mech. Eng..

[50] Jindal S., Manzoor F., Haslam N., Mancuso E. (2021). 3D Printed Composite Materials for Craniofacial Implants: Current Concepts, Challenges, and Future Directions. Int. J. Adv. Manuf. Technol..

[51] Jindal P., Bharti J., Gupta V., Dhami S.S. (2023). Mechanical behaviour of reconstructed defected skull with custom PEEK implant and titanium fixture plates under dynamic loading conditions using FEM. J. Mech. Behav. Biomed. Mater..

[52] Carpenter R.D., Klosterhoff B.S., Torstrick F.B., Foley K.T., Burkus J.K., Lee C.S., Gall K., Guldberg R.E., Safranski D.L. (2018). Effect of Porous Orthopaedic Implant Material and Structure on Load Sharing with Simulated Bone Ingrowth: A Finite Element Analysis Comparing Titanium and PEEK. J. Mech. Behav. Biomed. Mater..

[53] Sun C., Kang J., Yang C., Zheng J., Su Y., Dong E., Liu Y., Yao S., Shi C., Pang H. (2022). Additive Manufactured Polyether-Ether-Ketone Implants for Orthopaedic Applications: A Narrative Review. Biomater. Transl..

[54] Lambrecht J.T., Nyffeler T., Linder M. (2012). Thermal Conduction of Titanium Implants Under CO2 Laser Irradiation In Vitro. Ann. Maxillofac. Surg..

[55] Binhammer A., Jakubowski J., Antonyshyn O., Binhammer P. (2020). Comparative Cost-Effectiveness of Cranioplasty Implants. Plast. Surg..

